# The Emerging Role of MicroRNAs and Other Non-Coding RNAs in Cancer Cachexia

**DOI:** 10.3390/cancers12041004

**Published:** 2020-04-19

**Authors:** Joana M. O. Santos, Sara Peixoto da Silva, Rui M. Gil da Costa, Rui Medeiros

**Affiliations:** 1 Molecular Oncology and Viral Pathology Group, IPO Porto Research Center (CI-IPOP), Portuguese Oncology Institute of Porto (IPO Porto), 4200-072 Porto, Portugal; joana.oliveira.santos@ipoporto.min-saude.pt (J.M.O.S.); peixotodasilva.sara@gmail.com (S.P.d.S.); rmcosta@fe.up.pt (R.M.G.d.C.); 2 Faculty of Medicine of the University of Porto (FMUP), 4200-319 Porto, Portugal; 3 Center for the Research and Technology of Agro-Environmental and Biological Sciences (CITAB), University of Trás-os-Montes and Alto Douro (UTAD), 5001-911 Vila Real, Portugal; 4 Postgraduate Programme in Adult Health (PPGSAD), Tumour and DNA Biobank (BTMA), Federal University of Maranhão (UFMA), 65080-805 São Luís, Brazil; 5 Laboratory for Process Engineering, Environment, Biotechnology and Energy (LEPABE), Faculty of Engineering of the University of Porto (FEUP), 4200-465 Porto, Portugal; 6 Research Department of the Portuguese League Against Cancer—Regional Nucleus of the North (Liga Portuguesa Contra o Cancro—Núcleo Regional do Norte), 4200-177 Porto, Portugal; 7 Virology Service, Portuguese Oncology Institute of Porto (IPO Porto), 4200-072 Porto, Portugal; 8 Biomedical Research Center (CEBIMED), Faculty of Health Sciences of the Fernando Pessoa University, 4249-004 Porto, Portugal

**Keywords:** cancer cachexia, muscle wasting, adipose tissue wasting, ncRNAs, microRNAs, lncRNAs, circRNAs, biomarkers, therapeutic targets

## Abstract

Cancer cachexia or wasting is a paraneoplastic syndrome characterized by systemic inflammation and an involuntary loss of body mass that cannot be reversed by normal nutritional support. This syndrome affects 50%–80% of cancer patients, depending on the tumor type and patient characteristics, and it is responsible for up to 20% of cancer deaths. MicroRNAs are a class of non-coding RNAs (ncRNAs) with 19 to 24 nucleotides in length of which the function is to regulate gene expression. In the last years, microRNAs and other ncRNAs have been demonstrated to have a crucial role in the pathogenesis of several diseases and clinical potential. Recently, ncRNAs have begun to be associated with cancer cachexia by modulating essential functions like the turnover of skeletal muscle and adipose tissue. Additionally, circulating microRNAs have been suggested as potential biomarkers for patients at risk of developing cancer cachexia. In this review article, we present recent data concerning the role of microRNAs and other ncRNAs in cancer cachexia pathogenesis and their possible clinical relevance.

## 1. Introduction

Cancer cachexia is defined by an ongoing loss of skeletal muscle mass, with or without loss of fat mass, that leads to progressive functional impairment and cannot be reversed by conventional nutritional support, according to an international consensus [1]. This syndrome leads to a major reduction in quality of life by causing severe fatigue, reduces the response to anticancer therapy, and consequently increases morbidity and mortality [2]. Commonly used chemotherapeutic agents can worsen cancer cachexia [3].

This cachexia syndrome is characterized by several key features such as the release of pro-cachectic cytokines and factors by the tumor and immune cells, skeletal and cardiac muscle wasting and atrophy, altered energy balance, alteration of homeostatic control in the central nervous system and adipose tissue depletion [2,4]. Several recent studies have been aiming to identify the molecular mechanisms involved in cancer cachexia, especially those related to muscle wasting [5,6]. The identification and characterization of molecular pathways leading to cachexia is crucial in order to better understand its pathogenesis as well as to identify new therapy targets and disease biomarkers.

Cancer cachexia affects 50%–80% of cancer patients and it is responsible for up to 20% of cancer deaths [6,7]. Nevertheless, the criteria used to define cachexia are still not consistent across studies, despite an international consensus has already defined its diagnostic criteria [1,2]. Regardless of the criteria applied, it is clear that some malignancies are more associated with cachexia than others, even though the incidence and degree of cachexia will vary among patients with specific types of cancer [2,8]. For instance, about 85% of pancreatic cancer patients become cachectic but 15% do not, which may be due to variations in tumor phenotype or host characteristics such as the genotype [8]. Therefore, it is hard to predict which patients will develop cancer cachexia. Moreover, cancer cachexia is composed of three distinct stages: pre-cachexia, cachexia and refractory cachexia which is the last stage [9]. Refractory cachexia is the stage that is most frequently recognized by medical doctors since the early diagnosis of cancer cachexia is barely inexistent [9]. Additionally, it is also the stage at which interventions are the least likely to be effective; therefore, interventions should be initiated in the early stages of the syndrome [9,10]. Thus, biomarkers for cancer cachexia are urgently needed in order to early identify those patients most likely to suffer from cancer cachexia. Unfortunately, the biomarkers that have been proposed until now are far from universal and are still not ready for translation into clinical settings [6,7]. Additionally, some of the proposed biomarkers are proteins (e.g., pro-inflammatory cytokines and products from muscle degradation) and detecting proteins with biomarker potential may be expensive and time-consuming because of their complex structures and necessity to find the accurate detection methods or producing new antibodies [7,11]. On the other hand, nucleic acids biomarkers (e.g., microRNAs) require less time and lower costs, since nowadays the technologies that are used to detect nucleic acids can be easily performed [11].

It has been pointed out that the most effective way to treat cancer cachexia is to cure the cancer [12,13]. Unfortunately, this is not always possible, namely in certain cancer types strongly associated with cachexia (e.g., pancreatic cancer) [12]. On the other hand, experimental data shows that targeted ablation of pro-cachexia signaling pathways allows for extended survival even in the face of continuing tumor growth [2,14,15]. Moreover, cachexia increases the risk of treatment-related complications and toxicities, increases susceptibility to infections, causes fatigue, and increases hospitalization time [16,17]. Thus, it is crucial to treat cancer cachexia in order to improve patient’s survival and quality of life. Several therapeutic interventions have been tested, but the therapeutic options for this syndrome remain poor and very limited. This may be partly because most clinical trials enroll patients that are already in a refractory cachexia stage [9]. Additionally, cachexia is a multifactorial syndrome and multimodal therapeutic may be more effective in cachectic patients [13].

In recent years, non-coding RNAs (ncRNAs) such as microRNAs (miRs) were shown to regulate signaling pathways involved in the pathogenesis of cancer cachexia [18,19]. Dysregulated expression of several miRs was observed in cachectic mice and in patients [20,21], suggesting their association with the different features of cachexia and their use as potential biomarkers or therapeutic targets [18,19]. The aims of this review are to summarize the recent data on the role of miRs and other ncRNAs in cancer cachexia and to discuss their potential applications.

## 2. MicroRNAs in Muscle Wasting

**Figure 1:**
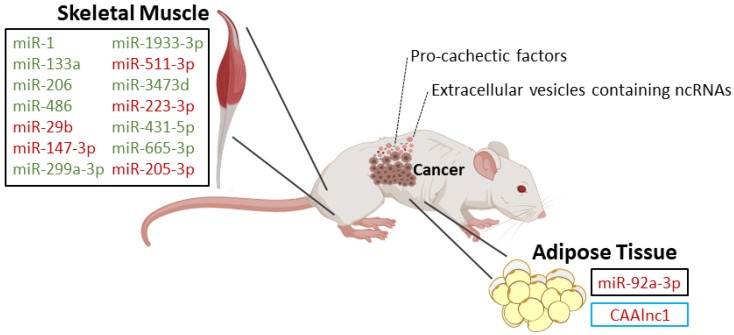
Dysregulated non-coding RNAs (ncRNAs) during muscle and adipose tissue wasting in mouse models of cancer cachexia: Squares in black refer to microRNAs and in blue refer to long non-coding RNAs. Upregulated non-coding RNAs are in red and downregulated non-coding RNAs are in green.

**Figure 2:**
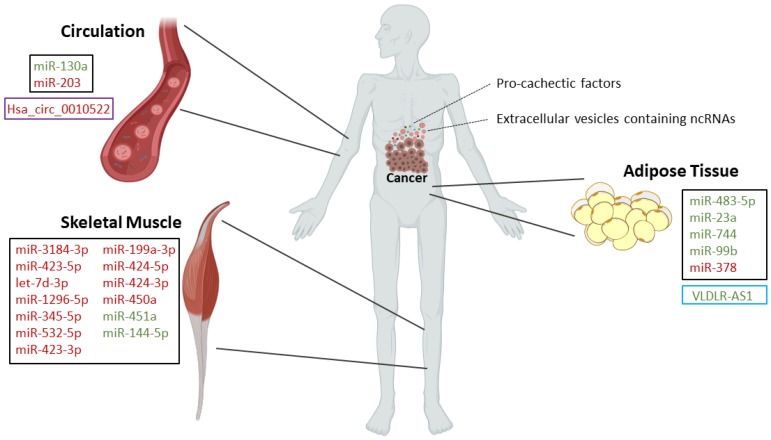
Circulating ncRNAs and dysregulated ncRNAs during muscle and adipose tissue wasting in cancer cachectic patients: Squares in black refer to microRNAs, in blue refer to long non-coding RNAs and in purple refer to circular RNAs. Upregulated non-coding RNAs are in red and downregulated non-coding RNAs are in green.

In vitro studies and studies in mouse models and humans have implicated miRs in muscle wasting during cancer cachexia (Figure 1, Figure 2 and Table 1). One study showed that microvesicles overexpressing miR-21 from lung and pancreatic cancer cell lines induce apoptosis of skeletal muscle cells [22]. In primary myoblasts from toll-like receptor 7 (TLR7)^−/−^ mice incubated with conditioned medium from Lewis lung carcinoma cells, the cell death was significantly reduced when compared with myoblasts from TLR7^+/+^ incubated with the same conditioned medium [22]. When incubating myoblasts from TLR7^−/−^ mice with microvesicles prepared from sera of cachectic pancreatic cancer patients, the same protection from cell death was observed [22]. Moreover, the exogenous addition of miR-21 increased cell death in TLR7^+/+^ myoblasts when compared with TLR7^−/−^ myoblasts [22]. Interestingly, inhibitors of c-Jun N-terminal protein kinase (JNK) and p38 were effective at reducing myoblast apoptosis in the presence of microvesicles or miR-21 [22]. JNK and c-JUN were transiently induced in proliferating myoblasts under microvesicles and miR-21 exposure, whereas no significant change was observed on p38 activity [22]. Thus, this study supports that microvesicles containing miR-21 promote cell death of muscle myoblasts by activating TLR7 signaling downstream to JNK [22].

Wild-type and poly (ADP-ribose) polymerase (Parp)-1^−/−^ and Parp-2^−/−^ mice with lung cancer develop cachexia, which is associated with decreased in miR-1 expression in skeletal muscles (diaphragm and gastrocnemius) [23]. MiR-133a is also downregulated in the diaphragm and gastrocnemius of cachectic wild-type and Parp-2^−/−^ mice when compared with the respective control [23]. In Parp-1^−/−^ animals, miR-133a was only downregulated in the diaphragm [23]. MiR-206 expression levels were downregulated in the diaphragm of Parp-1^−/−^ and Parp-2^−/−^ cachectic mice and downregulated in both gastrocnemius and diaphragm of cachectic wild-type mice [23]. Expression of miR-486 was decreased in both diaphragm and gastrocnemius of cachectic wild-type and Parp-2^−/−^ mice compared to their respective control animals [23]. However, in cachectic Parp-1^−/−^ mice, there are no differences in miR-486 expression in any muscle when compared to the control group [23]. Thus, these miRs were all downregulated in both diaphragm and gastrocnemius in cachectic wild-type mice when compared with the non-cachectic wild-type mice [23]. Moreover, in the gastrocnemius muscle of lung cancer-cachectic mice, *Parp-1* inhibition through miR-133a, miR-206 and miR-486 seems to promote muscle proliferation and differentiation while *Parp-2* inhibition through miR-206 seems to promote muscle differentiation [23]. Additionally, in the diaphragm, the deletion of *Parp-1* in mice favored the expression of miR-486, whereas in Parp-2^−/−^ mice, it was not observed significant effects on miRs expression [23]. Overall, this study suggests that *Parp-1* rather than *Parp-2* inhibition seems to exert more beneficial effects on muscle-related miRs expression of cachectic limb muscles in this specific mouse model of lung cancer cachexia [23]. Importantly, the differential results for the diaphragm and gastrocnemius point out the site specificity of signaling pathways controlled by miRs involved in cancer cachexia.

In mice that developed cachexia associated with Lewis lung carcinoma, the tibialis anterior muscle was used for microRNA sequencing [20]. Nine miRNAs were found to be differentially expressed, specifically miR-147-3p, miR-299a-3p, miR-1933-3p, miR-511-3p, miR-3473d, miR-223-3p, miR-431-5p, miR-665-3p and miR-205-3p, which are involved in biological processes such as altered cell-to-cell signaling, cell development, cell growth and inflammatory response, among others [20].

Using next-generation sequencing, miRs were profiled in skeletal muscle tissue from cachectic and non-cachectic pancreatic and colorectal cancer patients [21]. Eight miRs were found to be upregulated in cachectic patients, namely miR-3184-3p, miR-423-5p, let-7d-3p, miR-1296-5p, miR-345-5p, miR-532-5p, miR-423-3p and miR-199a-3p [21]. Further, miR-3184-3p, let-7d-3p and miR-1296-5p were validated by qRT-PCR, and 191 messenger RNA (mRNA) targets were identified for the eight miRs [21]. These targets play important roles in adipogenesis, myogenesis, signal transduction pathways, inflammation and innate immune response [21]. Moreover, the miRs that were identified showed prognostic and predictive value [21].

In patients with advanced stage non-small cell lung cancer, 28 differentially expressed miRs were identified in the quadriceps (vastus lateralis) muscle [24]. The expression of the highest-ranked miRs was further confirmed, showing that miR-424-5p, miR-424-3p and miR-450a were significantly upregulated and that miR-451a and miR-144-5p were downregulated in the muscle of cachectic patients when compared to healthy controls [24]. To differentiate between non-small cell lung cancer patients and healthy controls, a random forest classifier using miR expression as predictors for cachexia yielded results significantly different from random chance (area under the curve (AUC) = 0.5); the combination of miR-424-3p and miR-450a-5p yielded an AUC = 0.79 (95% confidence interval 0.6–0.9). The addition of miR-144-5p increased the AUC to 0.85 (95% confidence interval 0.7–0.9) [24]. Using miRTarBase, 158 validated genes were identified as potentially targeted by those miRs, and overrepresented pathways include the interleukin 6, transforming growth factor beta (TGF-β), tumor necrosis factor alpha (TNF-α), insulin and phosphoinositide 3-Kinase (PI3K)–protein kinase B (Akt) signaling pathways [24].

Interestingly, miR-29b upregulation was observed in numerous in vivo muscle atrophy models and is suggested to drive skeletal muscle atrophy in response to various stimuli, through targeting the *insulin-like growth factor 1 (Igf-1)* and *Pi3k subunit p85 alpha (p85α)* [25].

Besides cancer, there are other diseases related with cachexia. A few studies concerning miRs and muscle wasting in these diseases have been published.

A study performed in rats with cardiac hypertrophy and failure that develop cachexia showed that miR-29b-3p, miR-132-3p, miR-27a-5p, miR-337-5p, miR-434-3p, miR-539-5p, miR-136-5p, miR-210-5p, miR-322-3p, miR-331-3p, miR-376c-3p, miR-29a-3p, miR-204-5p, miR-30d-3p, miR-146b-5p, miR-632, miR-214-3p and miR-489-3p are differentially expressed in the soleus muscle of the cardiac cachexia group versus matched negative controls [26]. Integrative analyses of miR and mRNA expression profiles showed that these miRs affect genes involved in proteasome protein degradation, extracellular matrix (ECM) organization, respiratory electron transport and citric acid cycle [26]. Interestingly, miR-29a-3p and miR-29b-3p showed several target mRNAs in common, such as the ones that encode ECM-related proteins [26]. In vitro studies showed that myotube cell cultures derived from C2C12 cells and transfected with miR-29b mimics had a significant reduction in myotube area, total protein concentration, number of myotubes, and *myosin heavy chain (Myh)7*, *Myh2*, *collagen type I alpha 1 (Col1a1)* and *Col3a1* transcript levels [26].

A streptozotocin-induced diabetic mouse model was used to investigate the role of miRs in diabetes-associated cachexia [27]. Adeno-associated viruses that overexpress the miR-23a∼27a∼24-2 precursor RNA were injected in the tibialis anterior muscle, leading to upregulated levels of miR-23a, miR-27a and phosphorylated Akt. Moreover, it also reduced the levels of forkhead box O1 (FoxO1), phosphatase and tensin homolog (Pten) proteins, *tripartite motif-containing 63 (Trim63)* and *F-box protein 32 (Fbxo32)* transcripts, myostatin mRNA and protein levels, and phosphorylated SMAD family member 2 and 3 (Smad2/3) in skeletal muscles [27]. Moreover, miR-23a and miR-27a upregulation attenuated the diabetes-induced reduction of muscle cross-sectional area and rescued muscle function [27]. Curiously, these mice also showed reduced renal fibrosis compared with untreated controls, along with decrease levels of phosphorylated Smad2/3, alpha smooth muscle actin, fibronectin and collagen in kidney [27]. Moreover, the expression of miR-23a and miR-27a was also increased in serum exosomes and in the kidneys [27]. Thus, overexpression of miR-23a/27a in muscle may prevent diabetes-induced muscle cachexia and may attenuate renal fibrosis lesions, suggesting a muscle–kidney crosstalk [27].

A study using in vitro data showed that miR-424-5p targets mRNAs that encoded proteins associated with protein synthesis and required for the Pol I RNA pre-initiation complex which is necessary for ribosomal RNA (rRNA) transcription [28]. In mice, the expression of an orthologue of miR-424-5p caused muscle wasting as well as reduction of rRNA levels [28]. In human patients with chronic obstructive pulmonary disease, increased miR-424-5p expression was associated with disease severity and muscle mass reduction [28]. Preoperative miR-424-5p expression in skeletal muscle in patients submitted to aortic surgery was associated with muscle loss over the following 7 days [28].

Taken together, these data support the key role of miRs in regulating key pathways involved in skeletal muscle growth (e.g., PI3K signaling). Some miRs, like miR-29b, seem to coordinate signaling networks at the center of muscle atrophy and deserve further investigation as possible biomarkers or therapeutic targets.

## 3. MicroRNAs in Adipose Tissue Depletion

Besides skeletal muscle atrophy, lipolysis and browning of white adipose tissue are also important characteristics of cancer cachexia [29].

An in vitro study by Wu et al. showed that exosomes secreted by breast cancer cells (4T1 cells) may trigger cancer cachexia through miR-155 [30]. Adipocytes co-cultivated with breast 4T1 cells showed increased expression of uncoupling protein 1 (UCP1) and dramatically reduced expression of peroxisome proliferator activated receptor gamma (PPARG) and phosphorylated (P)-PPARG, which are involved in lipid accumulation [30]. Phosphorylated extracellular signal-regulated kinase (ERK)1/2 was also downregulated and P-p38 was upregulated [30]. Moreover, mature muscle cells co-cultivated with breast cancer cells underwent cell death, myosin heavy chain 1 loss, myotube atrophy, increased UCP3 levels, overexpressed P-p38 and downregulated P-ERK1/2, PPARG and P-PPARG when compared with muscle cells cultivated alone [30]. These findings support the hypothesis that miRs contained in tumor-derived exosomes may promote adipocyte and muscle fiber catabolism [30]. MiR-155 was found to be upregulated in exosomes derived from 4T1 cells and targeted *PPARG* in adipocytes, promoting brown differentiation and remodeling adipocyte metabolism [30]. Interestingly, the tumor cells co-cultivated with adipocytes or muscle cells exhibited increased invasiveness [30].

In line with these findings, mice injected with exosomes derived from chronic myeloid leukemia cells (K562 cells) suffered significant loss of weight and body fat [31]. Through RNA sequencing, miR-92a-3p was found to be upregulated in both K562 cells and their exosomes [31]. Moreover, adipose-derived mesenchymal stem cells could uptake the exosomes prevenient from K562 cells and, in turn, suppress the adipogenic ability [31]. In particular, miR-92a-3p from exosomes was able to suppress adipogenic ability by decreasing *CCAAT enhancer binding protein alpha (Cebpα)* expression [31].

In abdominal subcutaneous adipose tissue from cachectic patients with gastrointestinal cancers, miR-483-5p, miR-23a, miR-744 and miR-99b were downregulated whereas miR-378 was significantly upregulated when compared with cancer patients without cachexia [32]. Moreover, there was a strong positive correlation between miR-378 expression and catecholamine-stimulated lipolysis [32]. In vitro, primary human adipocytes obtained from multiple donors were transfected with a miR-378 hairpin inhibitor leading to a significant downregulation of norepinephrine-induced lipolysis and downregulation of *hormone-sensitive lipase E (LIPE)*, *patatin like phospholipase domain containing 2 (PNPLA2)*, and *perilipin 1 (PLIN1)* [32].

Overall, these observations confirm that tumor-derived miRs are able to contribute to lipolysis, including miRs exported via exosomes (Table 2). These miRs may be quantified in peripheral blood in the future, adding to their interest as potential biomarkers.

## 4. MicroRNAs in Cachexia-Homeostatic Control

The hypothalamus is essential to control energy homeostasis (e.g., thermoregulation) and food intake [33,34]. Multiple mediators, including inflammatory cytokines, act over hypothalamic regions to control those processes [33,35]. During cachexia, the hypothalamus mediates an increase in energy expenditure and loss of appetite [34].

A study addressed how miRs might regulate hypothalamic function and influence the pathogenesis of cachexia [36]. In this study, the authors evaluated the hypothalamic and cortical transcriptomes of anx/anx mice, which spontaneously develop an anorexia–cachexia syndrome [36]. In the hypothalamus of anx/anx mice, the predicted targets (115 out of 153) of most of the tested miRs were preferentially upregulated [36]. Conversely, in anx/anx brain cortex samples, mRNAs predicted to be targeted by miRs were preferentially downregulated [36]. In the hypothalamus, a closer look identified the upregulation of miRISC complex-related genes, namely *DGCR8 microprocessor complex subunit (Dgcr8)*, *fragile X mental retardation 1 (Fmr1)* and *argonaute RISC catalytic subunit 2 (Ago2)*, *DEAD box polypeptide 6* (*Ddx6*), and *poly(A) binding protein cytoplasmic 1* (*Pabpc1*), suggesting an altered miR machinery in the hypothalamus of anx/anx mice and its involvement in hypothalamic changes leading to cachexia [36]. However, the precise mechanisms through which an altered miR machinery in the hypothalamus may promote anx/anx mice phenotype remain to be determined and should be addressed by additional studies [36]. Additionally, studies concerning the role of miRs in the homeostatic control using cancer cachexia models should be performed.

## 5. Circulating MicroRNAs

Besides the intracellular localization of miRs, they can also be present in the extracellular environment and in biological fluids such as plasma and serum. In these fluids, circulating miRs are in complex with RNA binding proteins or encapsulated in extracellular vesicles (e.g., exosomes) in order to avoid their degradation [37]. Accumulating evidence shows that circulating miRs act as signaling intermediaries between cells [37].

Importantly, circulating miRs have shown clinical potential as biomarkers for several diseases [38]. Circulating miRs may be useful to detect cachexia in its early stages and to monitor its development in cancer patients. A study in head and neck cancer patients quantified plasmatic miR-130a expression and found that patients with low miR-130a expression when compared with the ones with high expression of miR-130a had higher plasma concentration of TNF-α and risk of being classified as cachectic [39]. Moreover, low miR-130a allowed to distinguish cachectic patients from moderately or mildly malnourished patients (79.4% sensitivity and 80.8% specificity; AUC = 0.865 (0.759–0.936)) [39]. MiR-130a followed by high level of TNF-α in plasma for the detection of cachectic patients showed 83.3% sensitivity and 91.7% specificity (AUC = 0.931 (0.794–0.988)) [39]. When body weight loss was >5%, the nutritional assessment using the subjective global assessment (SGA) was significantly improved by miR-130a (88.6% sensitivity, 94.3% specificity, 93.9% positive predictive value, and 89.2% negative predictive value) [39]. These results suggest that miR-130a has clinical potential as a biomarker for prediction of cachexia previous to radiotherapy in head and neck cancer patients. Additionally, miR-130a can improve the accuracy of SGA for the diagnosis of cachexia [39].

Another study evaluated the expression of miR-203 in the serum of colorectal cancer patients and showed that miR-203 was significantly increased in patients with low psoas muscle mass index (PMI) when compared with high PMI patients [40]. Intramuscular adipose tissue content was not correlated with tissue or serum miR-203 expression [40]. Moreover, elevated serum miR-203 expression was an independent predictive factor for myopenia [40]. The authors suggested that serum miR-203 expression may have potential as a biomarker for predicting myopenia in colorectal cancer patients. In vitro studies showed that, in human skeletal muscle cells transfected with miR-203 mimics, cell proliferation was moderately suppressed, apoptosis rate significantly increased and the number of viable cells significantly decreased [40]. Additionally, *baculoviral IAP repeat containing 5 (BIRC5)* (inhibitor of apoptosis) was identified as a target of miR-203 [40].

In chronic obstructive pulmonary disease, miR-422a levels in plasma were associated with muscle wasting [41]. Plasmatic levels of miR-422a were negatively correlated with muscle strength, and this miR was highly expressed in muscle [41]. In vitro studies showed that miR-422a suppressed *SMAD4* expression and inhibited the TGF-β signaling pathway in muscle cells [41]. These in vitro observations suggested that this miR may confer resistance to catabolism in the muscle [41].

## 6. Other Non-Coding RNAs in Cancer Cachexia

### 6.1. Long Non-Coding RNAs

Long non-coding RNAs (lncRNAs) are RNAs longer than 200 nucleotides that do not encode proteins [42]. Compared to other classes of non-coding RNAs, this class comprises a wide range of sizes, shapes and functionalities [42]. It is known that lncRNAs can have several functions such as regulating and remodeling chromatin architecture, modulating transcriptional regulation, regulating nuclear bodies, mRNA turnover and translation, and interfering with post-translational modifications [42]. LncRNAs can influence mRNA turnover in several ways, such as acting as miR sponges and thus reducing their regulatory effects [42,43,44]. Accumulating evidence indicates that lncRNAs are mediators of several diseases, such as cancer and cachexia [45,46,47]. However, the involvement of lncRNAs in cancer cachexia (Figure 1; Figure 2) and its mechanisms have only been described in a few studies [48,49].

#### 6.1.1. LncRNAs in Muscle Wasting

Since a disturbed myogenesis is associated with cachexia, LncMyoD could have an association with cachexia [50]. LncMyoD is an lncRNA that is directly activated by myogenic differentiation 1 (MyoD) during myoblast differentiation [50]. When active, LncMyoD binds to IGF2-mRNA-binding protein 2 (IMP2) and negatively regulates IMP2-mediated translation of crucial genes for proliferation (*neuroblastoma ras oncogene* (*N-Ras*) and *myelocytomatosis oncogene (c-Myc)*) [50]. Gong et al. described that the knockdown of this lncRNA inhibits muscle differentiation by leading to a failure to exit the cell cycle [50]. Thus, LncMyoD may be a potential cachexia-associated lncRNA [50].

The lncRNA muscle anabolic regulator 1 (MAR1) was newly described and associated with muscle differentiation and regeneration [43]. This lncRNA was found to be highly expressed in mouse skeletal muscle and positively correlated with muscle differentiation and growth, both in vitro and in vivo [43]. LncRNA MAR1 acts as a sponge of miR-487b, regulating Wnt family member 5A (Wnt5a) and promoting muscle differentiation and regeneration [43]. The overexpression of MAR1 attenuated muscle atrophy, maintaining muscle mass and strength [43]. Thus, MAR1 was thought to act as a novel therapeutic target for treatment of muscle atrophy that can be associated with cancer cachexia [43].

LncIRS1 is another newly identified lncRNA found to be enriched in mouse skeletal muscle and is thought to work as a competing endogenous RNA [51]. This study showed that lncIRS1 can regulate myoblast proliferation and differentiation in vitro and can regulate muscle mass and muscle fiber cross-sectional area in vivo [51]. The IGF1-PI3K/Akt pathway controls protein synthesis and degradation, and knockdown of the insulin receptor substrate 1 (IRS) decreases IGF1, leading to weigh loss in mice [52,53]. LncIRS1 acts a sponge for miR-15 family to regulate IRS1 expression, and its overexpression activates the IGF1-PI3K/Akt signaling pathway, promoting IGF-1 expression and activating AKT phosphorylation [51]. Thus, this lncRNA promotes skeletal muscle cell proliferation and differentiation, increasing muscle mass and countering muscle atrophy [51].

Sun et al. identified another novel lncRNA, Atrolnc-1, remarkably elevated in atrophying muscles from cachectic mice with chronic kidney disease [54]. Atrolnc-1 promotes activation of the nuclear factor-κB (NF-κB) and increases the *tripartite motif-containing 63 (Trim63)* transcription, which stimulates protein degradation and atrophy in muscles [54]. Inhibition of Atrolnc-1 ameliorates muscle wasting in mice by suppressing *Trim63* expression, and the authors speculate that this lncRNA may have an initiating role in cachexia via NF-κB activation [54].

LncRNAs seem to play an important role in muscle wasting. However, it should be noted that none of these studies were conducted in models of cancer-associated cachexia. This field of research will certainly benefit from additional experiments using adequate models of cancer cachexia.

#### 6.1.2. LncRNAs in Adipose Tissue Depletion

In 2018, Liu et al. analyzed adipose tissue samples from patients with gastrointestinal cancers, with and without cachexia [49]. The authors observed the downregulation of VLDLR Antisense RNA 1 (VLDLR-AS1), which was associated with cachexia and adipose tissue loss [49]. The authors predicted that VLDLR-AS1 regulates golgin A3 (GOLGA3), dual specificity phosphatase 14 (DUSP14) and ubiquitin C-terminal hydrolase L1 (UCHL1) through interaction with miR-600 and GOLGA3, zinc finger protein 219 (ZNF219), ring finger protein 141 (RNF141) and calumenin (CALU) though interaction with miR-1224-3p [49].

Some lncRNAs implicated in adipose tissue biology were identified in cachectic mice injected with colon-26 adenocarcinoma (C26) cells. Among these, knockdown of the cachexia-related anti-adipogenesis lncRNA 1 (CAAlnc1) was found to promote adipogenesis [48]. CAAlnc1 was most enriched in the liver and was also found to be more abundant in brown than in white adipose tissue [48]. The authors suggested that CAAlnc1 suppresses adipogenesis by interacting with an RNA-binding protein required for adipogenesis (HuR), leading to loss of adipose tissue [48].

### 6.2. Circular RNAs

Circular RNAs (circRNAs) are a class of RNAs produced by circularization of specific exons through the covalent bond of the 3′ end of an exon to the 5′ end of an upstream exon [55]. CircRNAs have been categorized as non-coding RNAs although some studies reported that certain circRNAs may code proteins [56]. Additionally, it has been shown that circRNAs may function as miR sponges, since they possess many miR-binding sites [57].

It has been demonstrated that circRNAs play important roles in the development of human disease, but their role in cancer cachexia has been poorly investigated [58].

#### CircRNAs in Adipose Tissue Depletion

There is one study regarding the role of a circRNA in cancer cachexia, showing that exosomes from patients with gastric cancer have a higher expression of circRNA Hsa_circ_0010522 (ciRS-133) when compared with the control group [59]. The expression of this circRNA was also increased in tumor tissue when compared with adjacent tissue [59]. Moreover, in gastric cancer patients, the expression of ciRS-133 was positively linked with the mass of brown adipose tissue and body fat rate [59].

In vitro studies showed that miR-133 directly associates with ciRS-133 [59]. Additionally, exosomes from SGC7091 (gastric adenocarcinoma) cells had a higher expression ciRS-133, and when added into the medium of 3T3L1 cells, induced upregulation of PR-domain containing 16 (PRDM16) and UCP1 [59]. Overexpression of miR-133 suppressed PRDM16 and UCP1, while knockdown of miR-133 increased PRDM16 and UCP1 levels [59]. Moreover, exosomes from SGC7091 cells induced accelerated glucose consumption ratio and higher oxygen consumption rate and increased the maximal respiratory capacity in 3T3L1 cells [59].

In mice injected with SGC7901 cells overexpressing ciRS-133, the expression of ciRS-133 in both tumor tissue and serum exosomes as well as in inguinal adipose tissues was confirmed to be significantly upregulated [59]. These mice showed browning and reduced weight of the inguinal adipose tissue [59]. These findings suggest that ciRS-133 aggravates tumor cachexia, possibly via adipose tissue browning, and that this circRNA may be a potential target for therapeutic development [59].

## 7. Conclusions

Cancer cachexia is caused by factors (e.g., inflammatory mediators and proteolysis-inducing factor) that are released by the tumor and by the host immune response to the presence of the tumor [4,6]. Recently, some of those factors have been identified as miRs and other ncRNAs, and their emerging role in the pathogenesis of cancer cachexia is increasingly recognized [18,19]. Recent studies have uncovered the role of miRs as well as some lncRNAs and circRNAs, mainly in muscle and adipose tissue wasting, using in vitro and/or in vivo approaches (Figure 1 and Figure 2). It should be emphasized that animal models are an important tool to study the role of ncRNAs in wasting, since the procedures for obtaining muscle/adipose tissue biopsies from human patients are invasive and therefore difficult to obtain. Moreover, animal models are also a useful tool to test the potential of miRs and other ncRNAs as therapeutic approaches. However, it should be noticed that the criteria used to diagnose cachexia in animal models are not always clear and should be better described in future research.

Systemic inflammation is the main driving factor of cancer cachexia because inflammatory mediators induce signaling pathways that lead to muscle/adipose tissue wasting and contribute to metabolic abnormalities and activation of anorexigenic pathways [6]. NcRNAs like Atrolnc-1 were found to induce NF-κB activation, with major implications for cachexia. Bearing this in mind, the reciprocal regulation of ncRNAs and pro-inflammatory signaling pathways in cancer cachexia should be further explored.

Additionally, studies exploring the potential of circulating miRs as biomarkers remain scarce. This may be a promising field of research and future studies should help improve the early diagnostic and adequate monitoring of cancer cachectic patients. Moreover, since the presence of weight loss is a key feature of cachexia, ncRNA predictors of weight loss associated with cancer cachexia should be further explored.

Studies concerning lncRNAs and circRNAs in cancer cachexia are also scarce, with most research still focused on miRs. Overall, the biological roles of ncRNAs in cancer cachexia are still poorly explored. Additional studies are necessary in order to clarify their potential applications in clinical practice.

## Figures and Tables

**Table 1:** MicroRNAs (miRs) involved in muscle wasting.

Type of Study	MicroRNA	Expression	Targets	Biological Significance
*In vitro: myoblasts from TLR7*^−/−^*and TLR7^+/+^ mice* [22]	miR-21 [22]	Overexpressed in microvesicles secreted by lung and pancreatic cancer cell lines [22]	-	TLR7^+/+^ myoblast cell death [22]
In vivo: wild-type, Parp-1^−/−^ and Parp-2^−/−^ mice with and without lung cancer and cachexia [23]	miR-1 [23]	Downregulated in both diaphragm and gastrocnemius in all cachectic models [23]	-	These miRs are involved in biological process such as myoblast proliferation, hypertrophy, cell differentiation, and innervation [23]. Differential results for the diaphragm and gastrocnemius point out the site specificity of signaling pathways controlled by miRs involved in cancer cachexia [23].
	miR-133a [23]	Downregulated in diaphragm of all cachectic models and in gastrocnemius of Parp-2^−/−^ and wild-type cachectic mice [23]		
	miR-206 [23]	Downregulated in diaphragm of all cachectic models and in gastrocnemius of wild-type cachectic mice [23]		
	miR-486 [23]	Downregulated in diaphragm and gastrocnemius of Parp-2^−/−^ and wild-type cachectic micev [23]		
In vivo: tibialis anterior muscle from mice that developed cachexia associated with Lewis lung carcinoma [20]	miR-147-3p [20]	Upregulated [20]	-	Altered cell-to-cell signaling, cell development, cell growth, and inflammatory response [20]
	miR-299a-3p [20]	Downregulated [20]		
	miR-1933-3p [20]	Downregulated [20]		
	miR-511-3p [20]	Upregulated [20]		
	miR-3473d [20]	Downregulated [20]		
	miR-223-3p [20]	Upregulated [20]		
	miR-431-5p [20]	Downregulated [20]		
	miR-665-3p [20]	Downregulated [20]		
	miR-205-3p [20]	Upregulated [20]		
In vivo: rectus abdominis from pancreatic and colorectal cancer patients [21]	miR-3184-3p [21]	Upregulated [21]	-	Roles in adipogenesis, myogenesis, signal transduction pathways, inflammation, and innate immune response [21]
	miR-423-5p [21]	Upregulated [21]		
	let-7d-3p [21]	Upregulated [21]		
	miR-1296-5p [21]	Upregulated [21]		
	miR-345-5p [21]	Upregulated [21]		
	miR-532-5p [21]	Upregulated [21]		
	miR-423-3p [21]	Upregulated [21]		
	miR-199a-3p [21]	Upregulated [21]		
In vivo: quadriceps (vastus lateralis) muscle from non-small cell lung cancer patients [24]	miR-424-5p [24]	Upregulated [24]	-	Roles in interleukin 6, TGF-β, TNF-α, insulin, and PI3K-Akt signaling pathways [24]
	miR-424-3p [24]	Upregulated [24]		
	miR-450a [24]	Upregulated [24]		
	miR-451a [24]	Downregulated [24]		
	miR-144-5p [24]	Downregulated [24]		
In vivo/In vitro: gastrocnemius from numerous muscle atrophy models, including mice inoculated with mouse colon cancer C26 cells/C2C12 cells [25]	miR-29b [25]	Upregulated [25]	*Igf-1* and *Pi3k (p85)* [25]	To drive skeletal muscle atrophy [25]

TGF-β, transforming growth factor beta; TNF-α, tumor necrosis factor alpha; PI3K-Akt, phosphoinositide 3-Kinase (PI3K)–protein kinase B (Akt).

**Table 2:** MicroRNAs (miRs) involved in adipose tissue depletion.

Type of Study	MicroRNA	Expression	Targets	Biological Significance
In vitro: 3T3-L1 cell line [30]	miR-155 [30]	Upregulated in exosomes from breast cancer cells (4T1 cell line) [30]	*PPARG* [30]	Promotes brown differentiation and remodels adipocyte metabolism [30]
In vivo/In vitro: mice injected with K562 cells-derived exosomes/adipose-derived mesenchymal stem cells obtained from patients [31]	miR-92a-3p [31]	Upregulated in exosomes from chronic myeloid leukemia cells (K562 cells) [31]	*Cebpα* [31]	Loss of body fat in mice and suppression of the adipogenic ability of adipose-derived mesenchymal stem cells [31]
In vivo/In vitro: Abdominal subcutaneous adipose tissue from cachectic patients with gastrointestinal cancers/primary human adipocytes [32]	miR-483-5p [32]	Downregulated [32]	-	MiR-378 enhances adipocyte lipolysis [32]
	miR-23a [32]	Downregulated [32]	-	
	miR-744 [32]	Downregulated [32]	-	
	miR-99b [32]	Downregulated [32]	-	
	miR-378 [32]	Upregulated [32]	-	
